# Case of Varicose Aneurism, Sucessfully Treated by Pressure

**Published:** 1846-12

**Authors:** Wm. Johnston

**Affiliations:** White-house, N. J.


*Case of Varicose Aneurism, sucessfully treated by Pressure.* By Wm. Johnston, M. D., of White-house, N. J.—Jacob Apgar, a blacksmith aged 78, consulted me on the 4th of March last on account of an injury received in blood-letting about four weeks previously. Upon uncovering his arm I discovered an aneurismal varix about the size of a walnut, produced by a wound of the brachial artery. Its pulsation was synchronous with the artery at the wrist. There had been very extensive effusion of blood in the cellular membrane for several inches, both up and down the arm; but this had disappeared after a few days, and at the time I now examined the arm there was no discoloration of the tumour, which presented a slight irregularity over its surface. Its pulsations were so strong as to raise the fingers with a bound, when pressed firmly upon it; indeed, the artery at the wrist pulsated with unusual force. The impulse of arterial blood into the aneurismal sac immediately under the cicatrice in the vein was very striking, and left no manner of doubt as to the real nature of the case. He complained of no pain upon handling the tumour. His general health was good, though his habits had been rather intemperate. He stated to me that there had been some difficulty in stopping the bleeding, and that when his attention was first directed to the tumour it was about the size of a hazelnut.

I was much struck in reading the relation of several cases of spontaneous aneurism successfully treated by pressure, and resolved to give the plan a trial. I had an arched tourniquet constructed so as to touch the arm, only at two opposite points. It was made of steel. At one end there were a couple of holes for screwing on a piece of board of three or four inches in diameter, which was well padded, and at the other end a long screw, which was attached to a similar board, on the back of which was a female screw. The boards were suited to the convexity of the arm. The apparatus was placed upon the most convenient part of the humerus to command the artery. By means of the screw I could regulate the pressure upon the artery so as instantly to stop the circulation in it, or merely weaken its force. The first application of the instrument, which was continued less than two hours, completely destroyed the *aneurismal bruit;* I could not detect it afterwards. The circulation through the artery was completely interrupted about one-third of the time in which the instrument was applied. *My principal object, however, was merely to weaken greatly the force of the circulation in the artery of the arm and thus to produce a state favourable to the coagulation of blood in the aneurismal tumour, and consequent deposition of fibrine.* The instrument was applied again on the next day for the space of less than two hours. Upon the following morning I found that all pulsation had ceased in the tumour, and the most careful examination could detect it in no part thereof. It never returned. The instrument was applied on the six following days and on every alternate day until the 23d of the month, when it was laid aside, and the patient advised to keep his arm at rest in a sling. The tumour became harder; almost of a cartilaginous feel, but slowly decreased in size, and at the time of writing this, June 26, (3 mo. 22 days,) is about as large as a split pea, and in all probability at the end of a few months more there will be no vestige of disease remaining. He has the free and perfect use of his arm. The instrument was not left on the arm after the first two applications longer than one hour at a time, and in reflecting upon the case I am satisfied that it would have terminated favourably had the instrument been applied but twice. Coagulation of blood in the sac, and deposition of fibrine and interruption to the ingress of arterial blood were in all probability effected, and nothing was wanting but patience to wait the progress of absorption.—*American Journal of Medical Sciences.*

